# Human placentation: foundations and implications for reproductive endocrinology and infertility

**DOI:** 10.1080/19396368.2025.2533992

**Published:** 2025-08-01

**Authors:** Elakkiya Prabaharan, D. Randall Armant, Sascha Drewlo

**Affiliations:** a Department of Obstetrics and Gynaecology, Sunnybrook Research Institute, University of Toronto, Toronto, Canada;; b Department of Obstetrics and Gynecology, Wayne State University School of Medicine, Detroit, MI, USA

**Keywords:** Placenta, trophoblast, perinatal disorders, assisted reproduction

## Abstract

Human fetal development requires sustenance *via* the placenta, which mediates molecular transport between maternal and fetal circulations. Placental formation begins as cells of the trophoblast lineage differentiate and the extraembryonic mesoderm becomes vascularized, assembling a unique organ *de novo* that facilitates nutrient and gas exchange, waste removal, hormone production and immune modulation. We describe how placentation is orchestrated to keep pace with fetal growth, but is vulnerable to disruption by medical interventions for infertility. Initially, trophoblast stem cells differentiate into proliferating mononuclear cytotrophoblasts (CTBs) that fuse to form the multinucleated syncytiotrophoblast (STB). The STB ensheathes the chorionic villi, bathed in maternal blood. As fetal blood vessels develop within the mesodermal core of villi, the maternal-fetal interface is established. Where the villi meet the decidua, CTBs further differentiate into extravillous trophoblasts, which invade and remodel uterine arteries into high-conductance, low-resistance vessels, enhancing maternal blood flow to the placenta. Among the critical intercellular axes that govern trophoblast differentiation, invasion, and vascular remodeling hormonal cues, particularly those associated with the corpus luteum, are critical; their alteration in certain assisted reproductive technology (ART) protocols can contribute to incomplete arterial remodeling. Malplacentation is linked to miscarriage, fetal growth restriction, and preeclampsia, affecting over 10% of pregnancies, and occurring at higher rates in patients diagnosed with infertility, especially those who conceive through ART. Understanding the mechanisms driving these pathologies is essential for improving pregnancy outcomes. Strategies to optimize ART protocols and therapeutic interventions targeting key signaling pathways offer potential avenues to mitigate risks associated with malplacentation.

## Introduction

**Figure 1.**
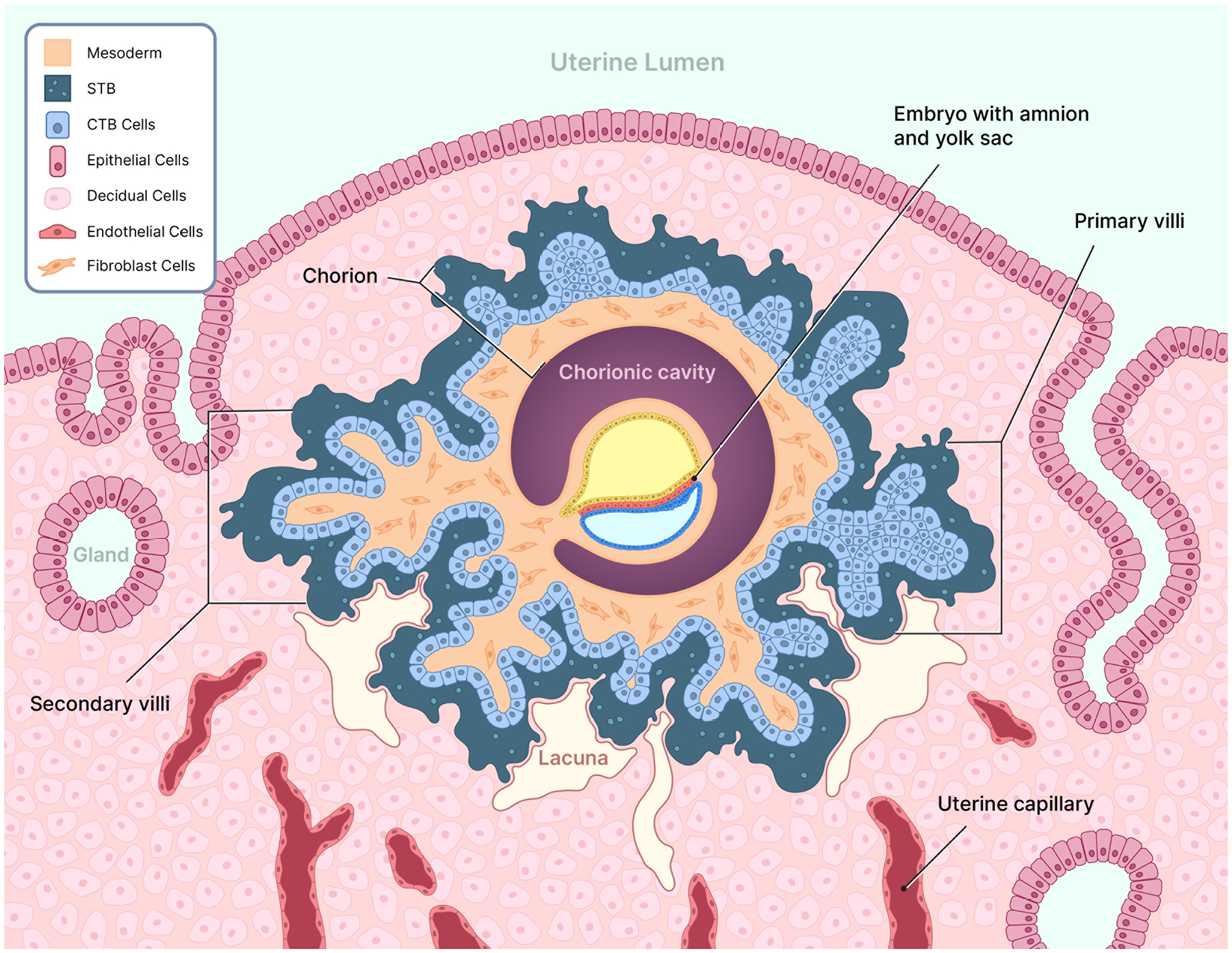
Embryo implanted in the upper portion of the uterine decidua at embryonic days 11–16. Mononuclear cytotrophoblast (CTB) cells have fused to form invading syncytiotrophoblast (STB) that consumes uterine decidua, glands and capillaries. The uterine epithelium and decidua have regenerated the tissue overlying the conceptus that has implanted interstitially. A mixture of maternal blood plasma infiltrating from capillaries and the contents of glands disrupted by STB accumulates in cavities within the decidua, creating lacunae. As the embryo grows and invades deeper into the decidua, CTB cells underlying the STB proliferate, forming primary chorionic villi that protrude into the uterine tissue. Extraembryonic mesoderm spreads over the amnion and yolk sac and across the basement membrane of CTB cells circumventing the chorionic cavity. As mesoderm migrates into villi under the CTB layer, secondary villi are formed. The mesoderm, CTB and STB located outside the chorionic cavity comprise the chorion. Note: the embryo is drawn to illustrate structures that form throughout the second week of embryonic development, but not necessarily at the same time (e.g., primary and secondary villi appear sequentially). Illustration adapted from Norwitz et al. (2001).

The placenta is a unique and vital organ that develops during pregnancy, serving as the interface between the mother and developing embryo. This temporary organ facilitates the exchange of nutrients, gases, and waste products, produces hormones essential for maintaining pregnancy, and modulates immune tolerance of the semi-allogenic fetus. Placental development commences shortly after implantation of the blastocyst into the decidualized uterine lining (Benirschke et al. 2006). Trophoblast cells, derived from the embryonic trophectoderm, undergo syncytialization and engulf maternal structures comprising decidual tissue, glands, and blood vessels (Figure 1). During implantation, the trophoblast differentiates into two subtypes: cytotrophoblast (CTB) and the syncytiotrophoblast (STB). The inner layer of mononuclear CTB cells divide, differentiate, and fuse to form the STB, a multinucleated cell mass comprising the outer layer of the chorionic villi that directly facilitates nutrient, gas and waste exchange between the embryo and mother.

Placental pathologies, designated collectively as malplacentation, arise when deficiencies occur at the maternal-fetal interface that impede efficient exchange. Understanding placental development is crucial in the practice of reproductive endocrinology and infertility. Individuals undergoing assisted reproductive technologies (ART), such as intrauterine insemination or *in vitro* fertilization (IVF), have higher rates of malplacentation (Omani-Samani et al. 2020; Chih et al. 2021; Galanti et al. 2024), leading to significant pregnancy complications that include preeclampsia (PE), fetal growth restriction (FGR), miscarriage, and stillbirth.

Placental development is governed by a complex regulatory system involving numerous signaling pathways, transcription factors, hormonal influences, and environmental factors (Knöfler et al. 2019). This chapter will focus on several key pathways that play critical roles in trophoblast differentiation, invasion, and vascular remodeling. We will also examine how hormonal variations, particularly those associated with ART protocols, can impact placental development. Exploring these essential mechanisms will provide insights into their relevance for infertility and ART, highlighting how disruptions to these pathways can lead to pregnancy complications.

## Structure and formation of the chorion

**Figure 2.**
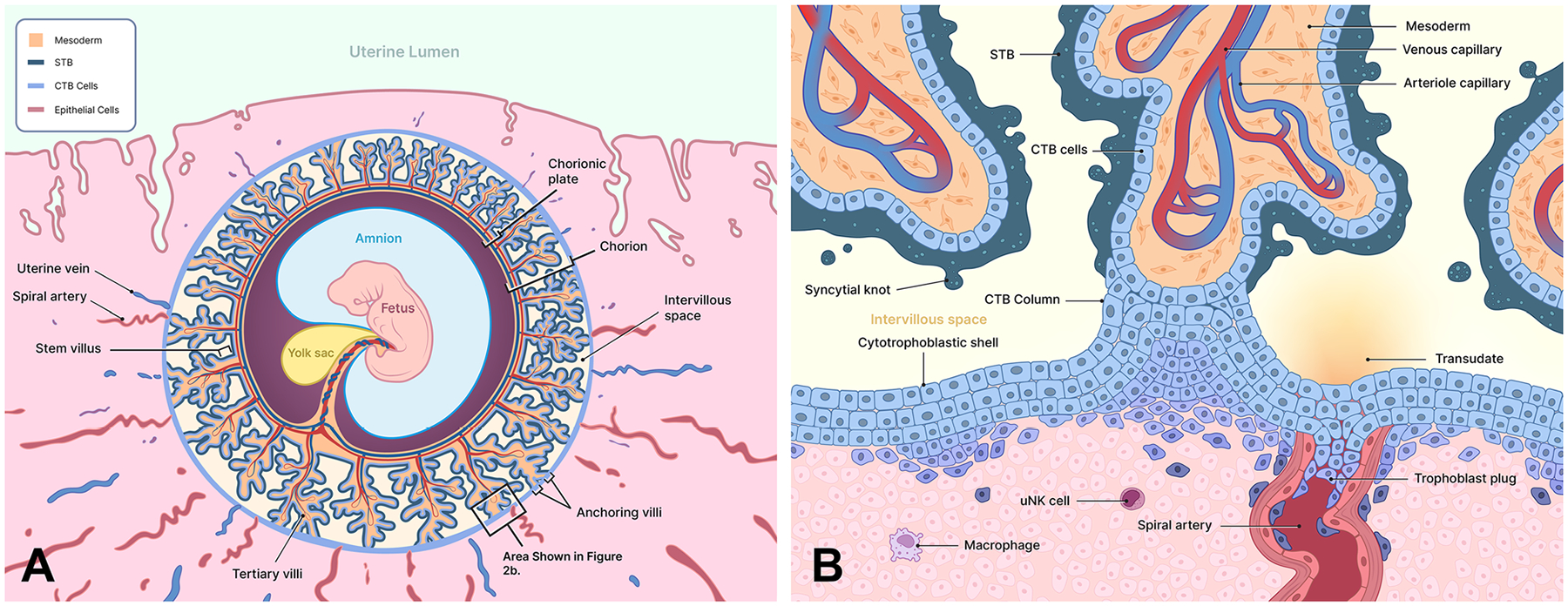
Development of the chorion into the early placenta. (**A)** The chorionic villi advance to tertiary villi as a fetal capillary system develops within the mesoderm beginning at embryonic day 21. Angiogenesis produces fetal blood vessels throughout the extraembryonic mesoderm during the first trimester, carrying blood from villous capillaries into the stem villi and across the chorionic plate to the umbilical cord, thus, completing the fetal circulation. The villus chorion, distal to the uterine cavity, will form the placenta, while villi proximal to and underlying the uterine lumen will eventually atrophy and become the avascular smooth chorionic membrane. The lacunae surrounding the chorion have coalesced to create a continuous intervillous space. Anchoring villi appear where the branching villi extend to and contact the decidua. (**B)** Shown in detail is an anchoring villus structure (area indicated in A). The tertiary villi, containing fetal arterioles (blue, deoxygenated) and venous capillaries (red, oxygenated), are modified at the point of contact with the decidua. The multinucleated syncytiotrophoblast (STB), producing syncytial knots of apoptotic nuclei that are shed into the intervillous space, is absent from the anchoring villi. The cytotrophoblast (CTB) cells are highly proliferative, generating a large column of cells (CTB cell columns) that spreads laterally across the surface of the decidua to adjacent anchoring villi, forming the cytotrophoblastic shell. Additionally, the CTB cells enter and occlude the spiral arteries to create trophoblast plugs. Trophoblast plugs restrict the high-pressure flow of blood emerging from the spiral arteries, which could otherwise generate sheer forces that would damage the early placental structures. Instead, blood filters through the CTB cells, with only a plasma transudate entering the intervillous space during the first 10 weeks of gestation. The most distal CTB cells will differentiate into extravillous trophoblast (EVT) cells that invade interstitially throughout the decidua or become endovascular to remodel the maternal arteries, beginning with disruption of the trophoblast plugs. EVT function is influenced by immune cells present in the decidua, including macrophages and uterine natural killer (uNK) cells. Illustrations adapted from Knöfler et al. (2019) and Aplin et al. (2020).

By the end of the fourth week of gestation (28 days after the beginning of the last menstrual period; embryonic day 14), the foundation of the placenta is laid with the formation of primitive utero-placental structures. Primitive STB aggressively invades the maternal decidual tissue while simultaneously expanding around the conceptus (Figure 1). As the STB penetrates deep into the decidual lining, it erodes maternal capillaries (sinusoids) and endometrial glands, creating small cavities within the stromal mass known as lacunae (Figure 1) (Benirschke et al. 2006). Initially filled with maternal plasma filtrate and uterine glandular secretions, the developing lacunar network provides essential nourishment and exchange to the embryo *via* diffusion through the STB. Simultaneously, mononuclear CTB cells, the proliferative progenitor population of the trophoblast, form a cellular layer protruding into the STB (Figure 1). The formation of primary chorionic villi begins as CTB cells proliferate and the overlying STB projects into the decidual tissue and lacunar network (Benirschke et al. 2006). As lacunae enlarge and coalesce, they will become the intervillous space, which is the primary site of maternal-fetal exchange (Figure 2A).

Progressing into the third week of embryonic development (days 15–21), extraembryonic mesodermal cells invade the core of the primary villi, transforming them into secondary villi (Figure 1) (Benirschke et al. 2006). The mesenchymal cores provide structural support and initiate the formation of the placental mesenchymal stroma, essential for placental architecture. Within the stroma, fetal macrophages known as Hofbauer cells emerge, contributing to immune regulation and villous development. By embryonic days 18–21, mesenchymal cells within the secondary villi differentiate into fetal capillaries, establishing the foundation of fetal circulation (Benirschke et al. 2006). This marks the formation of tertiary villi (Figure 2A), which contain blood vessels contiguous with the embryonic circulatory system to complete maternal-fetal exchange.

Trophoblast cells differentiate along two pathways: the villous trophoblast, comprising CTB and STB that generate the chorionic villi, and extravillous trophoblast (EVT) cells that invade the maternal decidua and modify the uterine spiral arteries. As the tertiary chorionic villi expand and branch, they form an intricate network resembling a tree-like structure, the villous tree (Figure 2A). Large stem villi connect directly to the chorionic plate, while branching villi extend into the intervillous space. CTB cells at the distal tips of villi contacting the decidua (Figure 2B) proliferate extensively to form CTB cell columns (Benirschke et al. 2006). These columns, composed of CTB cells without an overlying STB layer, attach the placenta to the maternal decidua basalis and are known as the anchoring villi. CTB cells from adjacent anchoring villi spread laterally and merge to create a continuous cellular layer across the decidua known as the cytotrophoblastic shell (Benirschke et al. 2006). It provides structural integrity to the placenta and regulates the depth of trophoblast invasion.

Proliferating CTBs in the cell columns of the anchoring villi and across the cytotrophoblastic shell form trophoblast plugs (Figure 2B) that occlude maternal spiral arteries throughout the decidual basalis (Jaffe et al. 1997). During most of the first trimester, trophoblast plugs within the spiral arteries limit the flow of maternal blood into the intervillous space, protecting the delicate placenta and embryo from potentially damaging shear stress caused by the entry of high-pressure arterial blood flow. In the presence of trophoblast plugs, only a plasma transudate of the blood filters through CTBs into the intervillous space under very low pressure (Burton and Jauniaux 2004).

CTB cells proliferate more rapidly under low oxygen concentrations (Genbacev et al. 1996), at levels similar to those in the intervillous space during the first trimester. These low oxygen levels enhance the rate of trophoblast proliferation, supporting the expansion of the occluding plugs. As a result, the limited force of arterial perfusion helps maintain the structural integrity of the placenta and produces a relatively hypoxic environment throughout much of the first trimester (Burton and Jauniaux 2004). At higher oxygen concentrations, CTB cells switch off proliferation and differentiate into invasive EVT cells (Genbacev et al. 1996), which first occurs in the most distal regions of the plugs where there is direct contact with oxygenated maternal blood. By weeks 10–12 of pregnancy, the trophoblast plugs are gradually eroded by emerging EVT cells until finally dislodged by arterial blood pressure. At that point, maternal blood freely enters the intervillous space to perfuse the chorionic villi. The onset of placental perfusion by maternal blood aligns with the strengthening and maturation of the placental villous structures and benefits the growing metabolic demands of the fetus.

**Figure 3.**
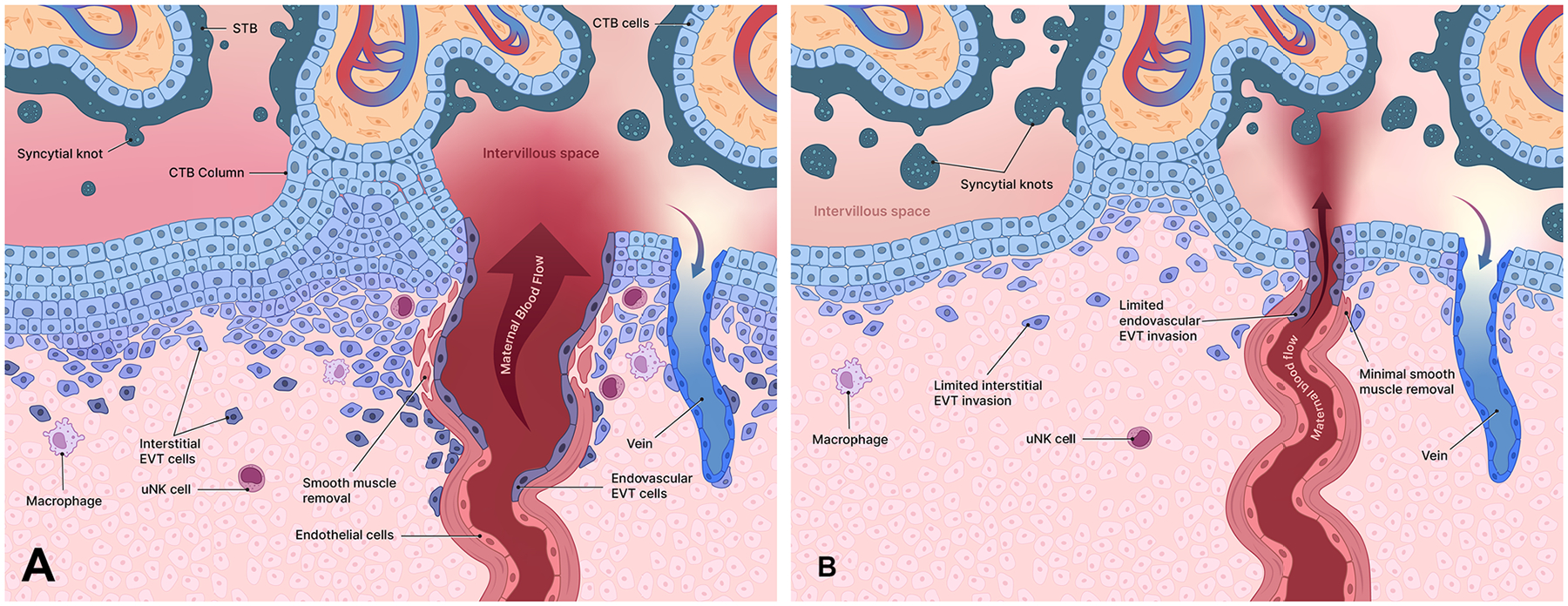
Remodeling of the uterine spiral arteries by invading extravillous trophoblast (EVT) cells emerging from the anchoring villi in normal development (A) and malplacentation (B). By week 10 of gestation, interstitial EVT cells have differentiated in the distal segment of the trophoblast column and migrate throughout the decidua where they contact immune cells, including macrophages and uterine natural killer (uNK) cells. (**A)** After dislodging the trophoblast plugs from the spiral arteries to initiate blood flow to the intervillous space, endovascular EVT cells remove the smooth muscle layer surrounding the arteries, converting them to permanently dilated vessels that pass high volumes of maternal blood into the intervillous space at relatively lower pressure. The arterial endothelial cells are removed by EVT cells that take on an endothelial-like phenotype and line the remodeled arteries. With these modifications, perfusion of the chorionic villi by maternal blood is increased without undue shear force as it enters the intervillous space. (**B)** In malplacentation, remodeling of the spiral arteries is incomplete. Interstitial and endovascular EVT invasion is shallow, leaving the distal arterial structures intact, including the smooth muscle layer. As a result, blood flow to the intervillous space is reduced, but at an elevated pressure. In the fetal compartment, the chorionic villi, subjected to damaging sheer stress and inadequate maternal blood perfusion, show signs of increased apoptosis, with heightened accumulation of syncytial knots. Illustrations adapted from Kingdom and Drewlo (2011).

The chorionic villi increasingly grow and branch as the STB expands through the fusion of underlying CTB cells (Benirschke et al. 2006). Trophoblast turnover involves the continuous renewal of the STB by the incorporation of new nuclei from fusing CTB cells and the removal of aging nuclei through apoptosis and shedding. This dynamic process maintains the functional integrity of the STB throughout gestation and is essential for placental function. During maturation of the placenta, syncytial sprouts—protrusions of the STB—extend from the surfaces of chorionic villi floating in the intervillous space (Benirschke et al. 2006). These structures contribute to the growth and branching of the villi, increasing the surface area for maternal-fetal exchange. Aging nuclei within the STB become apoptotic and aggregate to form structures known as syncytial knots (Figure 2B) that protrude from the villous surface (Burton and Jones 2009). Syncytial knots eventually detach from the surface of STB and are carried off into the intervillous space before entering the maternal circulation. The degradation of syncytial knots in the maternal bloodstream releases fragments of cell-free fetal DNA, which is utilized in non-invasive prenatal testing for detecting chromosomal abnormalities (Wong and Lo 2016; Guseh 2020). While the formation and shedding of syncytial knots are normal physiologic processes, an excessive increase in syncytial knots is associated with malplacentation (compare in Figure 3A,B) and the ensuing placental pathologies, such as PE (Burton and Jones 2009).

## Uteroplacental circulation

The uteroplacental circulation is fundamental to pregnancy, facilitating the exchange of gases, nutrients, and waste products between the maternal and fetal bloodstreams. This exchange occurs at the surface of the STB that ensheathes the chorionic villi (Knöfler et al. 2019). Maternal blood enters the intervillous space *via* the uterine arteries in the decidua basalis and bathes the chorionic villi, where maternal-fetal exchange occurs (Figure 3). Maternal blood depleted of nutrients and oxygen and now carrying fetal waste products exits the intervillous space through uterine veins (Knöfler et al. 2019).

As gestation progresses, the architecture of the chorionic villi adapts to increase the efficiency of exchange. Fetal capillaries within the villi progressively realign closer to the villus surface as mesodermal and CTB cells that surround them regress (Jones and Fox 1991). Internal remodeling of the villi reduces the distance between fetal and maternal blood, ultimately leaving only the fetal capillary endothelial cells and the STB to separate the two circulations (Jones and Fox 1991). These changes facilitate faster transfer of gases, nutrients, and waste products between mother and fetus (Huppertz 2023). Simultaneously, placental growth increases the number of villi and their branches, greatly expanding the available surface area of the STB to maximize exchange capacity and meet the growing metabolic demands of the fetus (Karimu and Burton 1995). By term, the surface area of the STB reaches approximately 12–14 square meters (Clavero and Botella 1963), illustrating the remarkable growth and adaptation of the placenta.

These adaptations in the villi coincide with changes in maternal blood flow. As pregnancy advances into the latter half of the first trimester, EVTs dislodge the trophoblast plugs, allowing maternal blood to flow freely from remodeled spiral arteries at low pressure and high volume, generating more capacity for maternal-fetal exchange (Roberts et al. 2017). At that time, the placenta has matured sufficiently to handle the increased oxygen levels without incurring oxidative damage (Burton et al. 2009a). Maternal spiral arteries that extend through the uterine muscle layer (myometrium) to the decidua undergo significant remodeling by EVT cells during early pregnancy (Figure 3A). These modifications greatly increase the volume and rate of maternal blood delivery into the intervillous space for perfusion of the chorionic villi. There are two main subtypes of EVTs involved in this process:

- Interstitial EVTs (iEVTs): These cells invade the decidual stroma and interact with maternal stromal cells, macrophages, and uterine natural killer (uNK) cells (Figures 2 and 3) (Clavero and Botella 1963). These interactions are crucial for regulating trophoblast invasion and modulating the maternal immune response to support successful implantation and placental development.
- Endovascular EVTs (eEVTs): These cells penetrate the spiral arteries, replacing the endothelial lining and removing the smooth muscle layer (Figure 3A), thereby transforming the vessels into permanently dilated, high-flow volume, low-resistance channels (Knöfler et al. 2019). Arterial remodeling increases maternal blood flow to the placenta, while reducing the velocity and sheer force of blood entering the intervillous space once the trophoblast plugs are dislodged.

These vascular changes occur in a physiologically hypoxic environment that is characteristic of the first trimester. Low oxygen tension influences trophoblast behavior through the stabilization of hypoxia-inducible factors (HIFs), particularly HIF-1α. HIFs act as transcription factors that regulate genes involved in:

- Trophoblast Proliferation and Differentiation: Hypoxia promotes the proliferation of CTB cells for villous growth and accumulation of trophoblast plugs, facilitating proper placental development (Caniggia et al. 2000; Wakeland et al. 2017).
- Chorionic Villus Development: HIF-mediated pathways support the early branching morphogenesis of villi, essential for expanding the surface area for exchange (Dunwoodie 2009; Nakamura et al. 2021).
- Vasculogenesis and Angiogenesis: Low oxygen levels induce the expression of angiogenic factors, including those in the vascular endothelial growth factor (VEGF) family, to promote the formation of new blood vessels within the placenta and establish a robust fetal circulation (Liu et al. 1995; Forsythe et al. 1996).
- Trophoblast Turnover: Hypoxia-induced pathways may also regulate the turnover of trophoblast cells, ensuring a balance between cell proliferation and apoptosis necessary to maintain a healthy cell population (Carmeliet et al. 1998; Burton and Jones 2009).

Concomitantly with trophoblast development, non-trophoblastic placental lineages emerge from the extraembryonic mesoderm. Pluripotent mesenchymal stem cells in the villous core differentiate into hemangioblasts, which give rise to angioblasts (endothelial precursors) and hematopoietic cells (Demir et al. 2007). Around day 21 of embryogenesis, endothelial tubes begin forming, with angiogenesis expanding the placental vasculature into a complex capillary network through the first trimester (Demir et al. 2007). Placental vasculogenesis and angiogenesis—alongside maternal vascular function—are tightly regulated by various pro- and anti-angiogenic factors, notably those in the VEGF family (Burton et al. 2009a). VEGF, produced by STBs, angiogenic precursors and Hofbauer cells, signals through paracrine interactions with its receptors, VEGFR-1/FLT1 and VEGFR-2/KDR, on precursor cells to drive vasculogenesis, angiogenesis and lymphangiogenesis (Demir et al. 2007). Placental growth factor (PlGF), another VEGF family member produced by CTBs and endothelial cells, promotes VEGF activity *via* FLT1 binding, enhancing KDR signalling. PlGF levels rise throughout pregnancy, and its dysregulation is linked to maternal vascular malperfusion and malplacentation (McLaughlin et al. 2020; 2021; Chaemsaithong et al. 2023). Placental hypoxia during the first trimester upregulates VEGF and FLT1 to promote angiogenesis (Shore et al. 1997), but persistent hypoxia due to poor spiral artery remodeling can disrupt angiogenic balance, reducing PlGF and increasing soluble FLT1 (sFLT1), which contributes to maternal endothelial dysfunction and PE (Maynard et al. 2003; 2008).

By the second and third trimesters, the mature placenta receives approximately 700 mL of maternal blood per minute, with about 150 mL of blood present in the intervillous space at any given time. As blood flows from the spiral arteries (70 mmHg) into the intervillous space (10 mmHg), the drop in pressure is optimal for maternal-fetal exchange and the structural preservation of the villi (Burton et al. 2009a; Gill et al. 2011). The coordination between trophoblast development, vascular remodeling, and oxygen tension illustrates the extensive adaptations necessary for the development of the utero-placental circulation.

## Impact of infertility and ART on placentation

It has become clear in recent years that women with fertility disorders who become pregnant spontaneously, as well as those who undergo ART therapeutic interventions to become pregnant, are at increased risk for pregnancy complications associated with aberrant placental development. Certain medical conditions that affect fertility can also increase the risk of adverse pregnancy outcomes. For example, conditions such as preexisting obesity, diabetes and chronic hypertension—often linked to infertility—are known risk factors for perinatal disorders (Kawwass and Badell 2018). There is evidence that an infertility diagnosis without ART intervention puts pregnancies at increased risk for low birthweight, small for gestation birthweight, gestational hypertension and perinatal death, while the risk increases further with ART. Studies of singleton pregnancies in which the same women diagnosed with infertility conceived one pregnancy without ART intervention and another with ART reveal increased risk of low birthweight and preterm birth in those pregnancies where ART was used (Romundstad et al. 2008; Henningsen et al. 2011; Dhalwani et al. 2016). Comparison of pregnancies of fertile women who each had a spontaneous singleton pregnancy and another in which they served as a surrogate carrying an ART-conceived pregnancy showed increased risk of low birthweight, gestational hypertension, gestational diabetes and preterm birth when ART was used (Woo et al. 2017).

It has been suggested that ART is responsible for a three-fold higher probability of low birthweight with several caveats that include multiple gestations, the choice of fresh or frozen embryo transfer, number of embryos transferred and endometrial preparation (Reig and Seli 2019). A meta-analysis of 15 studies examining singleton pregnancies that included 1.9 million spontaneously conceived and 12,283 IVF pregnancies found that the odds ratio was significantly higher in IVF pregnancies for small for gestational age, very low birthweight, low birthweight, preterm delivery and perinatal mortality. Further analysis showed a significantly higher prevalence of gestational diabetes and PE associated with IVF. Gestational hypertension and PE have shown up as significant risks in more recent meta-analyses, comparing pregnancies conceived spontaneously to those obtained through ART or IVF (Thomopoulos et al. 2017; Omani-Samani et al. 2020; Chih et al. 2021). These studies strongly suggest an increased risk for malplacentation and the resulting perinatal pathologies in pregnancies achieved using ART interventions to overcome infertility.

Individuals undergoing ART may experience alterations in placental development due to several factors. Although it has become rarer in recent years with adoption of single embryo transfer in IVF, the occurrence of twin or multiple gestation pregnancies in ART-conceived pregnancies increases the risk of FGR, gestational diabetes, hypertension and preterm birth (Kawwass and Badell 2018). The hormonal environment is significantly affected, as exogenous hormonal support may not fully replicate the natural hormonal milieu, potentially affecting trophoblast function and placentation (Csapo 1956). Ovarian hyperstimulation syndrome is commonly reported in association with ART treatments (Kawwass and Badell 2018). The absence of a functional corpus luteum in certain ART protocols that suppress ovulation reduce hormone and growth factor production required for placentation (von Versen-Höynck et al. 2019). The lack of corpus luteum-derived hormones, such as progesterone and relaxin, has been associated with higher rates of PE in ART-conceived pregnancies (von Versen-Höynck et al. 2019).

## Malplacentation: Mechanisms and implications in reproductive endocrinology

Malplacentation, affecting up to 10% of pregnancies, leads to serious complications such as FGR, PE and miscarriage, posing a significant risk to maternal and fetal health. These complications arise from disruptions in the intricate processes that establish a healthy maternal-fetal interface, including trophoblast invasion, survival, spiral artery remodeling, and hormonal regulation (Pijnenborg et al. 1983; Espinoza et al. 2006).

Abnormal spiral artery remodeling due to inadequate EVT invasion can have severe consequences for placentation and pregnancy outcomes. The development of a healthy placenta relies on the transformation of uterine spiral arteries into high-flow, low-resistance vessels, primarily driven by EVT activities (Figure 3A). In normal placentation, EVTs invade the maternal decidua and remodel uterine arteries, ensuring an adequate supply of maternal blood to the placenta (Benirschke et al. 2006). In malplacentation (Figure 3B), impaired EVT invasion and inadequate remodeling leave blood vessels in a high-resistance state that restricts blood flow to the placenta, increases blood pressure within the uterine arteries, and elevates the sheer force of blood spurting into the intervillous space (Burton et al. 2009b). As such, severe damage to the placenta by excessive sheer forces during the first trimester can lead to a miscarriage. Moreover, reduced perfusion of the chorionic villi due to impeded blood flow adversely alters placental function by reducing nutrient and waste exchange and creating a hypoxic environment in the second trimester (Genbacev et al. 1996; Caniggia et al. 2000). The heightened turbulence and restricted flow of maternal blood entering the intervillous space reduces villus perfusion, increases shear stress, and generates oxidative stress, ultimately compromising fetal development and contributing to perinatal pathologies.

Persistent hypoxia and oxygen free radical damage may further exacerbate already inadequate trophoblast differentiation, resulting in persistent high-resistance vessels and compromised villous function. This persistent state can lead to:

- Impaired Placental Development: Disruption of the normal maturation processes hampers the placenta’s ability to support fetal growth effectively.
- Oxidative Damage: Elevated levels of reactive oxygen species can cause cellular damage within the placenta, compromising its structural and functional integrity.
- Increased Risk of Pregnancy Complications: Conditions like PE and FGR are associated with poor placental perfusion and oxidative stress, posing risks to both maternal and fetal health (Cindrova-Davies and Sferruzzi-Perri 2022).

Therefore, the precise regulation of uteroplacental circulation, including the timing of increased maternal blood flow and the extent of spiral artery remodeling, is vital for normal placental and fetal development. The interrelationships between these mechanisms underscore the key role of early placental development in determining pregnancy outcomes.

The hormonal environment plays a crucial role in placental development. Hormones produced by the corpus luteum, such as relaxin and VEGF, facilitate proper trophoblast function and spiral artery remodeling. These activities support maternal cardiovascular adaption to pregnancy and healthy placental development (Conrad 2020; Niazi and Dumanski 2024). In ART protocols where embryo transfer is conducted without a natural or induced ovarian cycle (e.g., frozen or donor embryo transfer), the absence of corpus luteum-derived hormones and growth factors increases the risk of gestational hypertension and PE by impairing trophoblast invasion and spiral artery remodeling (Conrad et al. 2019; von Versen-Höynck et al. 2019). These hormonal imbalances disrupt critical signaling pathways, such as those involving glial cell missing homolog 1 (GCM1), which are essential for syncytialization and proper placental development (Chen et al. 2025).

Several key molecular pathways regulate placental development. The transcription factor GCM1 is pivotal for driving syncytialization, which is critical for maternal-fetal exchange and effective hormone production. Dysregulation of GCM1—whether through hormonal deficiencies, epigenetic changes or signaling disruptions—impairs syncytialization and contributes to malplacentation (Baczyk et al. 2009). Matrix metalloproteinases (MMPs) play a vital role in EVT invasion by degrading the extracellular matrix during spiral artery remodeling (Benirschke et al. 2006). Dysregulated MMP activity can exacerbate trophoblast dysfunction by failing to activate signaling pathways (Drewlo et al. 2011), as in the case of heparin-binding EGF-like growth factor (HBEGF), through MMP2-dependent growth factor shedding (Jain et al. 2017).

Malplacentation is a spectrum disorder in that, depending on the severity and structural impact on the placenta, the disease state can manifest as relatively mild (low birthweight, mild FGR, pregnancy-induced hypertension) to more serious illness (severe FGR, PE, preterm labor) to life-threatening disease (miscarriage, stillbirth, HELLP syndrome, and complications of severe PE that include thrombosis, placental abruption, eclampsia and maternal death) (Burton and Jauniaux 2004). Clinical and animal studies indicate there are multiple causes underlying these syndromes, particularly PE (Jung et al. 2022). Malplacentation alone does not completely account for all PE that is observed clinically or pathologically, suggesting a wider spectrum of etiologies (Huppertz 2018). The hypothesis that PE can be separated into two somewhat distinct etiologies is gaining support and proposes that they are associated with disease beginning either before 34 weeks (early-onset PE) or after reaching 34 weeks (late-onset PE) of gestation (Magee et al. 2022). Malplacentation plays a major role in early-onset PE, while late-onset PE, accounting for about 70% of PE, is associated with maternal factors (maternal cardiovascular maladaptation) that cause an imbalance in the uteroplacental circulation (e.g., macrosomia, multiple gestations, pre-existing maternal cardiovascular disease). This hypothesis is gaining support at the molecular level through systems biology technologies, but more research is needed.

Single-cell RNA sequencing (scRNAseq), which has greatly advanced understanding of the molecular landscape of the diverse cell types within the placenta (Liu et al. 2018; Suryawanshi et al. 2018; Vento-Tormo et al. 2018; Arutyunyan et al. 2023; Derisoud et al. 2024), analyzed in conjunction with circulating cell-free RNA signatures, was used to validate the utility of cell-free transcriptomics to track the trajectory of trophoblast differentiation in the context of early-onset PE (Tsang et al. 2017). The scRNAseq approach is being used to explore new cellular subtypes within the placenta and the molecular regulation that orchestrates placentation, as well as the etiological divergence of early- and late-onset PE (Solt et al. 2025).

A large multicenter study prospectively collected second trimester maternal blood for transcriptomic analysis of cell-free RNA to examine molecular subtypes and their relationship to malplacentation phenotypes in a design that used 5,399 specimens for training and 2,829 for validation (Elovitz et al. 2025), Clinical subgroups of gestational hypertension were distributed among eight categories that, based on their molecular subtypes, could be recombined into two groups that were diagnosed either before 37 weeks of gestation or after 37 weeks. The early-onset group was characterized by altered expression of placental-associated genes, while pregnancies with late-onset disease instead expressed immune-associated transcripts. These classifications, based on gene expression, align well with the clinical distinctions along the spectrum of placental derived perinatal disorders, as well as current understanding of the biology underlying disease heterogeneity.

There are limitations in systems biology approaches for understanding perinatal disease. With scRNAseq, placentas from patients with PE are only available after delivery, not during the period when the disease is developing. Transcriptomic analysis of circulating cell-free RNA has the advantage of accessing early stages of gestation but is limited by the dilution of placenta-derived RNA with contributions from all other organs. Certain populations of placental cells, particularly EVT cells and possibly other cell types, can be accessed noninvasively from the reproductive tract during ongoing pregnancies by collecting cervical mucus (Moser et al. 2018), providing opportunities to investigate their development and role in the early phases of malplacentation. Safely interrogating the placenta during pregnancy is a major challenge driving the innovation of new technologies.

## Endocrine functions of the placenta

The placenta serves as a pivotal endocrine organ during pregnancy, synthesizing and secreting a diverse array of hormones essential for maintaining gestation and supporting fetal development. These hormones orchestrate physiological changes in the mother, ensuring optimal conditions for fetal growth and preparing the body for parturition and beyond (Litwack 2022). We focus here on hormones that are physiologically significant for placental function, as well as those relevant in the context of ART.

### Peptide hormones

One of the primary peptide hormones produced by the embryo and placenta is human chorionic gonadotropin (hCG). Its secretion from the STB during the second week of embryonic development sustains the corpus luteum, ensuring continuous production of progesterone and preventing menstruation (Moore et al. 2020). Levels of hCG peak around the eighth week of gestation and subsequently decline as placental steroidogenesis and production of progesterone are established.

Another critical peptide hormone is human placental lactogen (hPL), also known as human chorionic somatomammotropin. hPL modulates maternal glucose and lipid metabolism, ensuring a steady supply of nutrients to the fetus (Burton and Jones 2009; Knöfler et al. 2019; Moore et al. 2020). Additionally, hPL promotes the growth and differentiation of mammary glands in preparation for lactation, facilitating the mother’s ability to nurse postpartum. Prolactin-related proteins further contribute to mammary gland development and lactation, working synergistically with hPL to prepare the breasts for milk production (Moore et al. 2020).

The placenta produces chorionic thyrotropin and corticotropin, which play significant roles in fetal development and the timing of birth, respectively. Chorionic thyrotropin stimulates fetal thyroid gland development, ensuring adequate production of thyroid hormones necessary for growth and neurological development (Moore et al. 2020). Corticotropin releasing hormone influences the timing of parturition by regulating the production of labor-inducing hormones and prostaglandins (Gibb and Challis 2002).

### Steroid hormones

Progesterone, a hormone vital to pregnancy, is initially synthesized by the corpus luteum. Progesterone production is transferred to the placenta around 7–9 weeks of gestation, utilizing maternal cholesterol as a substrate (Csapo 1956). Progesterone maintains uterine quiescence by inhibiting muscle contractions. It also supports endometrial receptivity and promotes immune tolerance of the fetus, ensuring that the maternal immune system does not reject the semi-allogeneic fetus (Csapo 1956).

Estrogens, predominantly estradiol (E2), are extensively produced by the placenta and play multiple roles during pregnancy. E2 promotes angiogenesis, facilitating the formation of new blood vessels and enhancing uteroplacental blood flow. It also mediates the vasodilatory effects of VEGF, contributing to increased uterine blood flow (Albrecht and Pepe 2010; Napso et al. 2018). Furthermore, estrogens induce changes in the maternal cardiovascular system, including increased cardiac output and blood volume, to meet the heightened metabolic demands of pregnancy.

The placenta also plays a crucial role in cortisol regulation through expression of 11β-hydroxysteroid dehydrogenase type 2, an enzyme that converts active cortisol to its inactive form, cortisone. This protective mechanism ensures that excessive maternal cortisol does not impair fetal growth and development (Csapo 1956; Coe and Lubach 2014).

### Growth hormones and insulin-like factors

Placental growth hormone, secreted by the STB, is instrumental in influencing maternal metabolism and fetal growth. It promotes insulin sensitivity in the mother and facilitates the transfer of nutrients to the fetus, ensuring sustained fetal growth (Moore et al. 2020). Additionally, the placenta produces insulin-like growth factors (IGFs), particularly IGF-1 and IGF-2, which are involved in cell proliferation, differentiation, and overall fetal growth. IGFs act in an autocrine and paracrine manner to regulate placental development and function, ensuring that the placenta adapts to the increasing needs of the growing fetus (Sferruzzi-Perri et al. 2011; Moore et al. 2020).

### Temporal changes in hormone production

The synthesis and secretion of placental hormones exhibits temporal variations throughout pregnancy (Aaron Geno et al. 2021). For example, hCG peaks in the first trimester to support early pregnancy by maintaining the corpus luteum and declines thereafter as progesterone and E2 production by the placenta takes precedence. E2 and progesterone levels continue to rise into the third trimester, prior to the onset of parturition when progesterone declines (Moore et al. 2020; Aaron Geno et al. 2021).

### Endocrine perturbation by ART

The endocrine functions of the placenta are particularly pertinent in the context of ART interventions in infertile women. Understanding placental hormone dynamics is crucial for optimizing ART outcomes and managing potential complications. ART procedures often involve the administration of exogenous hormones to induce ovulation and support early pregnancy. ART protocols aim to mimic the natural hormonal environment necessary for successful implantation and placental development (Huang and Rosenwaks 2012). For example, progesterone supplementation is frequently employed in ART-conceived pregnancies to stabilize the endometrium and maintain uterine quiescence, especially in cases where luteal phase support is inadequate due to ovarian stimulation protocols (Conrad and Baker 2013; Glujovsky et al. 2023).

Controlled ovarian hyperstimulation (COH) used in ART may lead to altered levels of placental hormones, potentially affecting placental function and fetal development. There is increased risk of low birthweight and FGR with IVF, but less so when embryo transfer is conducted in a cycle without COH, as with frozen or donor embryos (Kalra et al. 2011). Elevated E2 levels resulting from COH can negatively influence placental angiogenesis and vasodilation, impacting uteroplacental blood flow. These hormonal imbalances may predispose ART-conceived pregnancies to complications such as PE and FGR, necessitating more intense perinatal monitoring and management (Farhi et al. 2010; Imudia et al. 2012). Examination of programmed frozen embryo transfers in cycles where uterine receptivity is induced by exogenous E2 and progesterone revealed significantly lower average E2 levels in IVF patients with successful pregnancies and that pregnancy rates are negatively correlated with peak serum E2 concentrations (Fritz et al. 2017). Recent studies show that higher live birth rates and reduced early pregnancy losses are achieved when serum E2 levels are lower prior to progesterone administration in frozen embryo transfers (Li et al. 2022; Ozer et al. 2023; Shuai et al. 2024; Singh et al. 2024). E2 metabolism has been examined, demonstrating that during early pregnancy, E2 levels in women who later have PE are elevated 50% over those with normotensive pregnancies and there appears to be a general reduction in E2 metabolism (Cantonwine et al. 2019). These findings suggest that ART protocols that generate supraphysiologic E2 can contribute to malplacentation and associated adverse pregnancy outcomes.

It appears that, in addition to its other effects on pregnancy, elevated E2 negatively impacts the activity of EVT cells required for normal placentation. Examining the direct effect of E2 on trophoblast function, it was found in human placental villus explant cultures, as well as the EVT-like HTR-8/SVneo cell line, that exposure to E2 above concentrations that normally circulate during the first trimester specifically induces apoptosis and inhibits proliferation in a dose-dependent manner (Patel et al. 2015). Furthermore, trophoblast invasiveness was similarly inhibited by E2 in the cell line (Patel et al. 2015). Comparison of chorionic villus samples obtained in the first trimester from IVF pregnancies to villi from pregnancies that used non-IVF fertility treatment showed no differences in global DNA methylation, but did reveal several individual genes that were differentially methylated at multiple sites, including *CXCL14* that binds specifically to trophoblast cells and inhibits invasive activity through regulation of MMPs (Xu et al. 2017). Upstream analysis of the differentially methylated genes identified the trophoblast-specific (Paul et al. 2017) transcription factor GATA3 as a key regulator (Lee et al. 2016). GATA3 expression was inhibited by E2 in HTR-8/SVneo trophoblast cells, and knockdown of GATA3 suppressed cell migration and invasion (Lee et al. 2016). RNA sequencing with DEG and IPA analyses of GATA3 knockdown and wildtype HTR8/SVneo cell lines identified 200 differentially expressed transcripts that comprised cellular networks characteristic of trophoblast function, including cellular movement, cellular development, cell death and survival. These findings suggest one plausible mechanism in which E2 may disrupt the role of EVT cells in vascular remodeling, leading to malplacentation and related pregnancy disorders.

In IVF protocols with inadequate or absent corpus luteal support (von Versen-Höynck et al. 2019), there is a significant increase in the risk of malplacentation. The use of programmed cycles rather than natural cycles prior to transfer of frozen embryos has brought this problem to light (Singh et al. 2020). The corpus luteum, essential for endogenous progesterone production during the luteal phase and first trimester, also secretes vasoactive substances that aid in spiral artery remodeling. Without sufficient corpus luteal activity, placental development may be compromised, leading to high resistance uteroplacental circulation and hypoxic conditions, which are known risk factors for PE and FGR (von Versen-Höynck et al. 2019; Singh et al. 2020).

To mitigate these risks, ART protocols have been adjusted to ensure adequate luteal phase support. Strategies include extended progesterone supplementation beyond the typical luteal phase to support early placental development, the use of gonadotropin-releasing hormone agonists or antagonists to better control ovarian stimulation and preserve corpus luteum function, and individualized hormonal treatments tailored for specific patient profiles to enhance placental endocrine function and reduce the incidence of complications (Carp 2020; Ali and Mathur 2023). Monitoring placental hormones, such as hCG, E2 and progesterone, is vital to assess pregnancy viability and placental health in spontaneous pregnancies, and even more so in those conceived through ART. Levels of hCG and progesterone serve as biomarkers for early pregnancy viability, while abnormal hormone levels may indicate potential complications, allowing for timely intervention (Davies et al. 2003). Current evidence does not implicate *low* maternal hCG or progesterone in the genesis of PE or FGR. Conversely, several large cohort studies report that *elevated* second-trimester hCG concentrations (≥2.0–2.5 MoM) confer a 1.5- to 2-fold higher risk of PE and a modest increase in small-for-gestational-age births, although the latter effect wanes once PE is controlled for (Spong et al. 1998; Bartha et al. 2003; Skogler et al. 2023). Data for progesterone are equivocal; supraphysiologic late-pregnancy levels have been linked to subsequent PE, possibly *via* prostacyclin suppression (Moon et al. 2014), whereas low first-trimester levels chiefly predict miscarriage or defective decidualization rather than hypertensive placental disease.

## Immune functions of the placenta

Given the complexity of immune interactions during pregnancy, this section provides an overview of the key immune functions of the placenta, with a particular focus on how these functions relate to ART. The placenta employs multiple strategies to modulate the maternal immune system, ensuring the successful coexistence of the semi-allogenic fetus within the maternal environment, while maintaining the ability to defend against potential pathogens. This immunomodulatory function is essential for maintaining a healthy pregnancy and ensuring fetal development.

### Expression of non-classical MHC molecules

One of the primary mechanisms by which the placenta achieves immune tolerance is through the expression of non-classical major histocompatibility complex (MHC) class I molecules, such as human leukocyte antigen-G (HLA-G) and human leukocyte antigen-C (HLA-C) (Ferreira et al. 2017; Rouas-Freiss et al. 2021). Invasive EVTs prominently express HLA-G on their surface. Unlike classical MHC molecules, HLA-G has limited heterogeneity and interacts primarily with inhibitory receptors on maternal immune cells, such as KIR2DL4 on NK cells and ILT2 on T cells and antigen-presenting cells (Rajagopalan and Long 2012). These interactions dampen the cytotoxic activity of immune cells, thereby preventing the destruction of trophoblasts or other fetal cells that encounter the maternal immune system, fostering an environment conducive to fetal survival (Hunt et al. 2005).

### Cytokine and chemokine secretion

The placenta secretes a variety of cytokines and chemokines that further modulate immune cell activity, promoting a tolerogenic environment. Key cytokines such as interleukin-10 and transforming growth factor-beta (TGF-β) play pivotal roles in suppressing inflammatory responses and enhancing regulatory T cell populations (Thaxton and Sharma 2010; Yang et al. 2021; Horvat Mercnik et al. 2024). These cytokines inhibit the activation and proliferation of maternal T cells and NK cells, reducing the likelihood of an adverse immune response against fetal antigens. Additionally, chemokines like CCL2 and CXCL10 facilitate the recruitment and retention of immune cells that support immune tolerance, such as anti-inflammatory macrophages (Hamilton et al. 2013).

### Interactions with maternal immune cells

EVTs and other trophoblast cells engage in intricate interactions with various maternal immune cells (Figures 2 and 3), including uNK cells, macrophages and dendritic cells (DCs).

Uterine NK Cells: Unlike peripheral NK cells, uNK cells are abundant in the decidua and play a supportive role in pregnancy. They secrete factors such as VEGF and PlGF, which promote the formation of blood vessels and the remodeling of spiral arteries (Hanna et al. 2006). The interaction between HLA-G on EVTs and inhibitory receptors on uNK cells modulates their activity, preventing excessive cytotoxicity, while enhancing their role in vascular development (Rajagopalan and Long 1999; Tilburgs et al. 2015).

Macrophages: Placental macrophages, also known as decidual macrophages, predominantly exhibit an M2-like anti-inflammatory phenotype (Brown et al. 2014). These cells produce anti-inflammatory cytokines and growth factors that support tissue remodeling and placental development. They also aid in clearing apoptotic cells and debris to maintain a healthy decidual environment (Abrahams et al. 2004).

Dendritic Cells: DCs are involved in antigen presentation and the induction of immune tolerance. Placental DCs express low levels of co-stimulatory molecules, which reduces their ability to activate T cells effectively. This characteristic promotes the differentiation of regulatory T cells and the suppression of effector T cell responses, further contributing to fetal immune tolerance (Mahajan et al. 2024).

### Impact of ART on placental immune modulation

Dysregulation of placental immune functions can lead to pregnancy-related complications. For instance, inadequate expression of HLA-G or impaired cytokine signaling may result in insufficient immune tolerance, increasing the risk of FGR, PE and recurrent miscarriage (Tilburgs et al. 2015). Understanding these immune mechanisms is crucial for research towards developing therapeutic strategies to address such complications and improve pregnancy outcomes (Chiang et al. 2024).

ART procedures, particularly those involving ovarian stimulation, can alter the immunological environment of early pregnancy. COH induced during ART procedures may lead to elevated levels of estrogen and progesterone, which can influence placental development and immune interactions (Szekeres-Bartho et al. 1990; 2005; Yie et al. 2006; Nguyen et al. 2019). These hormonal changes can affect the expression of immune-modulatory molecules and the secretion of cytokines and chemokines, potentially disrupting the delicate balance required for immune tolerance. For example, exposure of uNK cells to estrogen *in vitro* increases their cell migration and angiogenic cytokine secretion, which is critical for angiogenesis in the uterine endometrium during the establishment of pregnancy (Gibson et al. 2015).

Consequently, ART pregnancies—especially those using hormone-programmed frozen-embryo transfer that lack a corpus luteum—show higher rates of fetal-growth restriction, PE and recurrent miscarriage (Omani-Samani et al. 2020). The absence of a corpus luteum, progesterone and relaxin skew early decidual immunity, impair spiral artery remodeling and heighten placental oxidative stress (von Versen-Höynck et al. 2019; Sacha et al. 2020; Conrad et al. 2024b, 2024a). This pro-inflammatory milieu erodes immune tolerance at the maternal–fetal interface, predisposing to hypertensive disorders and sub-optimal fetal development (Raunig et al. 2011; Conrad et al. 2019). Transcriptomic analysis of placental cell populations by scRNAseq indicates there is an immune dysregulation of cytokines that increases inflammation in early-onset PE, associated with the increased severity of maternal and fetal outcomes (Solt et al. 2025). These changes were minimal in late-onset PE, with preservation of immune regulatory mechanisms, aligning with the generally milder clinical manifestations.

### Therapeutic interventions and protocol adjustments

To mitigate the immune-related risks associated with ART, several therapeutic strategies have been proposed. These include optimizing ovarian stimulation protocols to preserve corpus luteum function, thereby ensuring adequate endogenous progesterone production and supporting immune tolerance (Singh et al. 2020). Additionally, targeted immunomodulatory treatments, such as the administration of anti-inflammatory cytokines or immune checkpoint inhibitors, are being explored to enhance placental immune function and reduce the incidence of complications like PE, FGR, and recurrent miscarriages (Conrad et al. 2024b). These interventions aim to restore the immune balance necessary for a successful and healthy pregnancy.

PE, in some cases, appears to involve cellular processes similar to aging, including STB oxidative stress and inflammatory disease (Redman et al. 2022). PE is characterized by an imbalance between pro-inflammatory (thromboxane A2) and anti-inflammatory (prostacyclin) factors. Low-dose aspirin is the only prophylactic agent with robust evidence for preventing PE, as its antiplatelet action restores the thromboxane-to-prostacyclin balance, reduces inflammation, and supports maternal systemic endothelial function (Sole et al. 2022). Initiating a daily aspirin regimen at 75–150 mg between 11 and 13 weeks of gestation (no later than 16 weeks) decreases the risk of early-onset PE, preterm birth, and FGR in high-risk women. The ASPRE (Combined Multimarker Screening and Randomized Patient Treatment with Aspirin for Evidence-Based Preeclampsia Prevention) randomized clinical trial reported a 62% fall in the incidence of PE prior to 37 weeks of gestation (Rolnik et al. 2017). Consequently, clinical guidelines recommend universal risk screening with targeted aspirin use.

In ART pregnancies, aspirin is often administered even earlier than 11 weeks to enhance uterine perfusion. However, the effect of early aspirin supplementation on implantation and live-birth rates remains inconsistent (Hurst et al. 2005; Grandone et al. 2014; Ghesquiere et al. 2022). Beyond aspirin, other immunomodulatory protocols, such as the administration of tacrolimus, are experimental. Tacrolimus can potentially mitigate maladaptive maternal-fetal immunity, but it carries a recognized association with gestational hypertension/PE, albeit lower than cyclosporine in some studies (Eugenia Rinella 2006; Louchet et al. 2024).

The benefits of empirical steroid regimens on placentation are also unclear. Supraphysiologic E2 at embryo transfer may itself impair placentation and raise PE amongst other risk factors (Chen et al. 2020; Sites et al. 2020; Stenqvist et al. 2025). Rigorous, adequately powered trials are required before incorporating these adjunct therapies into routine ART care.

### Monitoring and early detection

Effective monitoring of placental immune function in ART pregnancies could enable early detection and management of potential complications. The identification and use of biomarkers to assess HLA-G levels, cytokine profiles, and immune cell populations in the decidua is needed to provide valuable insights into the immune status of the placenta. The application of systems biology to identify immune regulatory molecules that are differentially expressed in normal vs malplacentation pregnancies could provide novel strategies for both diagnostics and new therapeutic approaches. Microarray studies of RNA from placentas of pregnancies with normal outcomes and PE suggest multiple etiologies that include an immunological origin (Leavey et al. 2016). Deeper analysis of the data revealed a secreted factor, fibrinogen-like protein 2, which is part of an immunoregulatory gene module that is differentially expressed in an immunological type of PE and oppositely in PE arising solely from malperfusion (Robineau-Charette et al. 2020). Early diagnosis of immune dysregulation would allow for timely intervention with appropriate immunogenic therapies, improving pregnancy outcomes and reducing the risk of adverse maternal and fetal health outcomes.

## Signaling pathways regulating trophoblast development

As previously discussed, trophoblast cells perform many essential functions during pregnancy, including nutrient and gas exchange, anchoring the placenta to the uterine mucosa, remodeling uterine spiral arteries and modulating vasculogenesis and angiogenesis to establish the uteroplacental circulation (Knofler 2010). The successful development of the placenta relies on early trophoblast proliferation and the precise differentiation of progenitors into specialized cell types. Investigating the signaling pathways that regulate trophoblast development can enhance our understanding of the mechanisms underlying normal placental formation, while unveiling potential causes of placental maldevelopment, particularly in the context of ART.

### Origin and differentiation of trophoblast cells

All trophoblast cells originate from the trophectoderm, the outer layer of the blastocyst. Despite significant progress in understanding placental development, the molecular mechanisms, particularly transcription factors (TFs), governing the transition from blastocyst implantation to the establishment of villi remain elusive. This knowledge gap arises partly due to limited access to human blastocysts and first-trimester placental tissue and the constraints on genetic studies involving these materials. Consequently, the developmental period of trophectoderm to cytotrophoblast (CTB) transition has yet to be fully explored. Trophoblast stem cells represent the earliest progenitor cells within the trophoblast lineage, possessing the capacity for self-renewal and differentiation into all trophoblast subtypes, including CTBs, STBs, and EVTs (Okae et al. 2018). CTBs are moderately differentiated progenitor cells that retain proliferative capabilities and serve as the source for STBs and EVTs within the placenta.

Over the last decade, several single-cell transcriptomics studies of trophoblast development have revealed various cell states and their lineages, as well as the differentiation pathways, signaling mechanisms, and cell-to-cell communication events that mediate early placental development. For instance, Vento-Tormo et al. (2018) identified several receptors that are upregulated during EVT differentiation, including *ACKR2*, which binds to inflammatory cytokines produced by maternal immune cells. Another group (Arutyunyan et al. 2023), combined scRNAseq with single-nuclei RNA sequencing to compare the regulatory programs driving EVT differentiation in both primary placental villi and placental organoids. Their study identified key transcription factors involved in EVT differentiation and described two previously uncharacterized intermediate EVT states (Arutyunyan et al. 2023). Collectively, these studies and numerous others provide a benchmark for designing experiments aimed at investigating defective EVT differentiation and invasion, as seen in pregnancy complications such as PE.

Omics techniques, such as scRNAseq, create large datasets from thousands of mRNA transcript readouts. This process can be highly erroneous and noisy. Genes that aren’t highly expressed can be missed, which may alter and misrepresent cell trajectory analyses, and variability between individual cells in a population may complicate the identification of discrete cell states (Heumos et al. 2023). Technical variations, such as batch effects, can confound results and be mistaken for new biological insights (Heumos et al. 2023). Moreover, validating the results of large-scale omics studies can be tedious and expensive. Despite these limitations, scRNAseq has provided significant advancements to trophoblast biology and an unprecedented look into the developmental processes dictating placental growth and function.

### Key transcription factors and established signaling pathways

GCM1 is a pivotal transcription factor that governs the differentiation of CTBs into STBs by regulating the expression of syncytin-1 and syncytin-2, fusogenic proteins essential for cell fusion during syncytialization (Pötgens et al. 2004). GCM1 also influences the differentiation of CTBs into EVTs, underscoring its multifaceted role in trophoblast development (Baczyk et al. 2005; Drewlo et al. 2009).

Peroxisome Proliferator-Activated Receptor Gamma (PPARγ) is a nuclear receptor involved in trophoblast differentiation, particularly in STB formation (Kadam et al. 2015). PPARγ regulates genes associated with lipid metabolism, energy homeostasis, and cell differentiation, which are crucial for the energy-intensive process of cell fusion. Additionally, PPARγ modulates GCM1 expression, thereby contributing to proper STB development (Levytska et al. 2014; Kadam et al. 2019; Armistead et al. 2021). Other transcriptional regulators, including cAMP-responsive element-binding protein, GATA-binding proteins, AP-2α and DLX3, also play significant roles in hormone expression and trophoblast differentiation (Knöfler et al. 2019).

HIF-1 regulates gene expression under hypoxic conditions, promoting Notch1 expression, which in turn influences EVT differentiation (Yu et al. 2019). The Notch signaling pathway is crucial for EVT behavior, including their invasive capacity and interaction with maternal tissues, thereby facilitating the remodeling of spiral arteries. Additionally, Wnt/β-catenin signaling is essential for regulating EVT proliferation and differentiation, ensuring that EVTs can effectively invade and remodel maternal spiral arteries (Knöfler and Pollheimer 2013; Knöfler et al. 2019).

Members of the Epidermal Growth Factor (EGF) family, including EGF, transforming growth factor alpha, and HBEGF, are expressed in trophoblasts throughout the placenta; however, reduced expression of these growth factors has been observed in placentas delivered from pregnancies with low birth weight and PE (Leach et al. 2002; Armant et al. 2015). These growth factors induce EVT differentiation *in vitro* (Leach et al. 2004), and autocrine HBEGF signaling protects trophoblasts from apoptosis when cultured at low (2%) oxygen comparable to that in the first trimester placenta (Leach et al. 2004). Given the increased apoptosis and reduced invasiveness of trophoblasts in placentas delivered by women with placental insufficiency (Brosens et al. 1972; DiFederico et al. 1999), the loss of EGF family signaling in perinatal disease may occur during early placentation and impact trophoblast survival and differentiation. In a baboon first-trimester model of placental insufficiency, HBEGF expression is significantly lower (Armant et al. 2020), along with reduced VEGF (Bonagura et al. 2012), in affected placentas. Placentas lacking the HBEGF gene, generated by mating heterozygous knockout mice, exhibit aberrant vasculogenesis and reduced levels of VEGF (Liu et al. 2019), suggesting that HBEGF drives essential VEGF expression. The low-oxygen environment of the placenta during the first trimester specifically elevates HBEGF in a trophoblast cell line through MMP2-mediated shedding of the HBEGF extracellular domain and downstream autocrine signaling that both increases HBEGF biosynthesis and inhibits trophoblast apoptosis (Armant et al. 2006; Jessmon et al. 2010; Jain et al. 2017). This positive feed-back loop could sustain CTB survival in the hypoxic environment induced by the occluding trophoblast plugs during the first ten weeks of gestation. Due to its affinity to heparan proteoglycans in the extracellular matrix, HBEGF primarily acts locally through autocrine and paracrine signaling, while EGF is readily secreted from the chorionic villi and enters the maternal circulation through venous drainage in the decidua basalis. EGF in maternal blood that contacts trophoblast cells in the distal region of the occluding plugs would, therefore, be expected to induce differentiation into invasive EVT cells that initiate erosion of the plug and arterial remodeling. Measurement of the serum EGF concentration at mid-pregnancy shows that levels in women who develop PE are significantly reduced to about half the level of control pregnancies (Armant et al. 2015). Therefore, aberrant EGF family signaling could contribute to the principal cellular pathologies observed with malplacentation, elevated apoptosis and reduced differentiation of the trophoblast.

### Interconnected signaling pathways and their regulation

The signaling pathways regulating trophoblast development are highly interconnected, creating a complex network that ensures precise control over trophoblast proliferation, differentiation, and invasion. For instance, cAMP/PKA signaling not only enhances GCM1 expression (Baczyk et al. 2009) but also interacts with Wnt/β-catenin and Notch pathways to coordinate EVT differentiation and function (Knöfler and Pollheimer 2013). PPARγ intersects with GCM1, integrating metabolic and growth factor signals to regulate trophoblast differentiation (Levytska et al. 2013a; Armistead et al. 2021).

An example of the intricate interplay between signaling pathways, transcription factors, and immunomodulatory factors in placental development is the lineage specification and differentiation of EVTs. A critical step in EVT lineage specification is the differentiation of proliferative progenitor CTBs into non-proliferative EVTs. The EVT lineage is first defined by the downregulation of CTB markers such as CD57 and Ki67, with upregulation of HLA-G and HLA-C (Pollheimer et al. 2018; Greenbaum et al. 2023). This transition is also marked by integrin switching, as CTBs downregulate integrin alpha (ITGA) 6 expression and increase ITGA5 and ITGA1 expression (Damsky et al. 1994; Chang et al. 2018; Pollheimer et al. 2018). Occurring in the distal portion of the CTB cell columns, this initial differentiation step occurs independently of the decidual environment. However, the chemokines, cytokines, and growth factors found in the decidual environment drive differentiation into iEVTs or eEVTs. For example, in an *in vitro* organoid model of the placenta, uNK cytokines induced the expression of both eEVT- and iEVT-specific markers in isolated EVTs, and markedly increased EVT migration out of the organoids. Another crucial step in EVT development is the upregulation of markers characteristic of the epithelial-mesenchymal transition (EMT) (Arutyunyan et al. 2023). Several studies have shown that Wnt signaling, *via* T-cell factor-4, controls EVT migration by promoting the expression of promigratory EMT genes (Knöfler and Pollheimer 2013). Finally, when EVTs differentiate into eEVTs and iEVTs, the identity of eEVT cells is marked by an upregulation of Notch signaling and downregulation of TGFβ signaling, whereas iEVT differentiation is characterized by TGFβ upregulation and Wnt inhibition (Arutyunyan et al. 2023).

Epigenetic modifications appear to fine tune these signaling pathways. DNA methylation and histone modifications can alter the expression of key transcription factors like GCM1 and PPARγ, thereby influencing trophoblast differentiation and function (Drewlo et al. 2021). Hormonal influences, particularly from estrogen and progesterone, modulate these transcription factors through receptor-mediated pathways, adding another layer of regulation (Knöfler and Pollheimer 2013).

### Impact of ART on trophoblast signaling

ART procedures, particularly those involving COH, can disrupt signaling pathways by altering the hormonal and epigenetic landscape of early pregnancy. For example, excessive E2 levels may over-stimulate certain pathways, causing premature differentiation or apoptosis of trophoblast cells, akin to a conductor misguiding an orchestra, resulting in a discordant performance. In baboons, shifting the normal surge in maternal serum E2 levels from the second to the first trimester of pregnancy suppresses EVT conversion of the spiral arteries, resulting in malplacentation and subsequent pathologies that resemble human FGR and PE (Aberdeen et al. 2012; Albrecht and Pepe 2020; Albrecht et al. 2021). This elevation of E2 during the period of uterine artery remodeling coincides with reductions in EGF and VEGF signaling (Bonagura et al. 2012; Armant et al. 2020). There is, in fact, evidence from human IVF that when ovulation induction excessively elevates E2 levels prior to fresh embryo transfer, there is increased risk of FGR and PE (Imudia et al. 2012). This study of 292 singleton IVF pregnancies examined peak serum E2 on the day of hCG, and compared pregnancies with the 90^th^ percentile highest levels (>3,450 pg/mL) to the remainder, finding a significantly higher proportion of pregnancies with FGR (3.8% vs 26.9%) and PE (4.5% vs 18.5%) in the elevated E2 group.

### Epigenetic modifications induced by ART

ART procedures can influence the epigenetic regulation of placental gene expression, modulating the expression of key immune-regulatory and signaling molecules, such as HLA-G, growth factors, transcription factors and cytokines, and thereby impacting the placenta’s ability to maintain immune tolerance and optimal trophoblast function (Chason et al. 2011; Nelissen et al. 2011; Drewlo et al. 2021). Epigenetic modifications have long-term implications for both maternal and fetal health (Barker 1995; Wadhwa et al. 2009) and trophoblast function (Chason et al. 2011), highlighting the necessity for careful monitoring in ART pregnancies. However, research to understand exactly how ART could modify the epigenetic regulation of placentation and its consequences is still at a very early stage.

## Clinical challenges and early detection

One of the greatest challenges in addressing malplacentation is the difficulty of identifying it early in pregnancy, particularly during the first trimester when effective interventions to restore trophoblast function are most likely to be beneficial. Current diagnostic tools, including Doppler ultrasound and serum biomarkers like PlGF and sFLT1, provide only indirect assessments of placental health. Unfortunately, these tools often detect dysfunction only after significant pathological changes have already occurred (Myatt et al. 2012), making early intervention challenging and increasing the likelihood that irreversible placental damage has occurred.

### Cervical trophoblasts: A novel diagnostic approach

A promising approach to early diagnosis of perinatal pathology is the interrogation of cervical trophoblasts that can be obtained as early as embryonic days 25–28 for assessing placental and fetal health (Bolnick et al. 2014). Hundreds of trophoblast cells that migrate from the placenta into the reproductive tract can be collected non-invasively, similar to a Pap procedure, offering unique insight into placental development (Drewlo and Armant 2017; Moser et al. 2018). By analyzing gene expression and molecular biomarkers of EVT cells, clinicians can detect early signs of malplacentation, allowing for timely and tailored interventions. Recent studies have demonstrated that cervical trophoblast analysis can effectively identify abnormal molecular expression patterns linked to placental dysfunction in the first trimester (Fritz et al. 2015; Bolnick et al. 2016; Kadam et al. 2019), making it a promising tool for identifying patients who would benefit from enhanced surveillance or early interventions, like low dose aspirin (Rolnik et al. 2017). This approach is particularly advantageous in ART-conceived pregnancies, where early detection is key to mitigating risks associated with placental insufficiency. The ability to monitor placental health non-invasively from the first trimester onward represents a transformative opportunity to implement targeted interventions, ultimately reducing the risk of adverse outcomes.

### Improving ART protocols to mitigate malplacentation

Understanding malplacentation is essential for improving pregnancy outcomes, particularly for ART-conceived pregnancies. Insights into the origins of placental insufficiency—including hormonal imbalances, disrupted signaling pathways, and inadequate trophoblast invasion—are pivotal for refining ART protocols. Ensuring adequate luteal phase support through progesterone supplementation or incorporating relaxin to mimic natural corpus luteum activity could mitigate risks associated with impaired placentation (von Versen-Höynck et al. 2019). Additionally, emerging therapeutic strategies—such as PPARγ agonists that promote trophoblast differentiation or low-molecular-weight heparin that supports angiogenesis—show promise for enhancing placental health and reducing complications.

The integration of novel diagnostic tools, such as cervical trophoblast analysis, with refined ART protocols represents a proactive approach to address the risk of perinatal disorders following ART. By enabling early identification and timely intervention, healthcare professionals can better support proper placental development, ultimately improving outcomes for both mothers and infants. A comprehensive understanding of placental biology, coupled with innovative diagnostic and therapeutic approaches, is crucial for advancing reproductive medicine and enhancing patient care. Continued research and proactive implementation of these strategies are essential for reducing adverse pregnancy outcomes globally.

## Improving pregnancy outcomes in ART

Optimizing ART protocols involves tailoring hormonal support to mimic natural corpus luteum hormonal patterns, including progesterone and potentially adding relaxin supplementation (Conrad and Baker 2013). Timing embryo transfers to align with the window of endometrial receptivity and using methods that minimize endometrial trauma are crucial. Refining culture media and conditions to reduce epigenetic alterations and support normal trophoblast differentiation and GCM1 expression may also improve outcomes.

Monitoring and intervention strategies, including early detection of key biomarkers (e.g., hCG, PlGF, sFLT1, EGF, HO1, and GCM1) could identify potential issues in the first trimester by monitoring placental function. Early diagnosis and new insights into the underlying mechanisms of malplacentation will help to develop novel therapeutic interventions. Possible medications for those at risk include PPARγ agonists to enhance trophoblast differentiation and invasion (Drewlo et al. 2011; Levytska et al. 2013b; 2014; Armistead et al. 2021; 2021; Grimaldi et al. 2022) and low-molecular-weight heparin to promote PlGF release from endothelial cells for restoring placental angiogenesis (McLaughlin et al. 2015, 2020, 2022). Lifestyle modifications, including advising patients on nutrition, stress management, and avoidance of environmental toxins, also support placental health.

## Future directions

A systems biology approach to investigating how ART affects placental immune functions can elucidate the mechanisms through which ART alters the immunological environment conducive to a successful pregnancy. By comparing and integrating multi-omics data, including the transcriptomic, epigenomic, proteomic, and metabolomic profiles of placentas between ART and ‘natural’ pregnancies, changes can be assessed in overall immunological tolerance. For example, transcriptomic analyses can determine whether ART procedures alter the expression of immune-related genes, such as the expression of non-classical MHC molecules by trophoblasts. Proteomic profiling can assess changes in the expression and secretion of immune mediators like cytokines and chemokines. Analyses of first-trimester placental villi using scRNAseq or assay for transposase-accessible chromatin with high-throughput sequencing (ATAC-seq) can map immune cell populations, including Hofbauer cells and uNKs, and their interactions with trophoblasts, which could provide insight into their altered functional states in ART pregnancies. Moreover, spatial biology approaches, including spatial transcriptomics, can provide insights into how ART procedures induce molecular changes that affect key processes like implantation, trophoblast invasion, and spiral artery remodeling.

After identifying key pathways and gene targets, it is important to connect these findings to real-world outcomes. As such, clinical and phenotypic data concerning complications like PE, FGR, and miscarriage, can be integrated alongside ART-specific variables such as embryo culture conditions, fertilization method (e.g., IVF), and fertility treatments (e.g., COH). Finally, RNAseq and spatial data sets can be used to identify immune pathways disrupted by ART, which can then be experimentally validated using *in vitro* models of placentation, such as organoids, trophoblast-uNK co-culture systems, and animal models.

## Abbreviations:

ART: Assisted reproductive technology
CBT: Cytotrophoblast
COH: Controlled ovarian hyperstimulation
DC: Dendritic cell
DEG: Differentially expressed gene
E2: Estradiol
eEVT: Endovascular EVT
EGF: Epidermal growth factor
EMT: Epithelial-mesenchymal transition
EVT: Extravillous trophoblast
FGR: Fetal growth restriction
FLT1: FMS-related receptor tyrosine kinase 1
GCM1: Glial cell missing homolog 1
HBEGF: Heparin-binding EGF-like growth factor
hCG: Human chorionic gonadotropin
HELLP: Hemolysis, elevated liver enzymes, low platelet count
HIF: Hypoxia-inducible factor
HLA: Human leukocyte antigen
hPL: Human placental lactogen
iEVT: Interstitial EVT
IGF: Insulin-like growth factor
IPA: Ingenuity pathway analysis
ITGA: Integrin subunit alpha
IVF: In vitro fertilization
KDR: Kinase insert domain receptor
MHC: Major histocompatibility complex
MMP: Matrix metalloproteinase
PE: Preeclampsia
PlGF: Placental growth factor
PPARγ: Peroxisome proliferator-activated receptor gamma
scRNAseq: Single-cell RNA sequencing
sFLT1: Soluble FLT1
STB: Syncytiotrophoblast
TGF-β: Transforming growth factor-β
uNK: Uterine natural killer
VEGF: Vascular endothelial growth factor
